# PAXIS: A Randomized, Double-Blind, Placebo-Controlled, Dose-Finding Phase 2 Study (Part 1) Followed by an Open-Label Period (Part 2) to Assess the Efficacy and Safety of Pacritinib in Patients with VEXAS Syndrome

**DOI:** 10.3390/jcm15041426

**Published:** 2026-02-11

**Authors:** David B. Beck, Maël Heiblig, Sinisa Savic, Marcela A. Ferrada, Arsène Mekinian, Onima Chowdhury, Danielle Hammond, Lachelle D. Weeks, Carmelo Gurnari, Yohei Kirino, Sophie Georgin-Lavialle, Sarah A. Buckley, Raman Garcha, Bryan G. Harder, Matthew J. Koster

**Affiliations:** 1 Center for Human Genetics and Genomics, NYU School of Medicine, New York, NY 10016, USA; 2 Hématologie Clinique, Lyon-Sud Hospital, Hospices Civils de Lyon, 69495 Pierre-Bénite, France; mael.heiblig@chu-lyon.fr; 3 Université Claude Bernard, 69100 Lyon, France; 4 Leeds Institute of Rheumatic and Musculoskeletal Medicine, University of Leeds, Leeds LS2 9JT, UK; s.savic@leeds.ac.uk; 5 Department of Medicine, University of Maryland School of Medicine, Baltimore, MD 21201, USA; mferrada@som.umaryland.edu; 6 Sorbonne Université, Service de Médecine Interne, AP-HP, Hôpital Saint-Antoine, CEREMAIAA, 75012 Paris, France; arsene.mekinian@aphp.fr; 7 Department of Haematology, Oxford University Hospitals’ NHS Foundation Trust, Oxford OX3 7LE, UK; onima.chowdhury@ndcls.ox.ac.uk; 8 Weatherall Institute of Molecular Medicine, University of Oxford, Oxford OX3 9DS, UK; 9 Department of Leukemia, The University of Texas MD Anderson Cancer Center, Houston, TX 77030, USA; dhammond@mdanderson.org; 10 Department of Medical Oncology, Division of Population Sciences, Dana Farber Cancer Institute, Boston, MA 02215, USA; lachelle_weeks@dfci.harvard.edu; 11 Department of Biomedicine and Prevention, University of Rome Tor Vergata, 00133 Rome, Italy; carmelogurnari31@gmail.com; 12 Translational Hematology and Oncology Research Department, Taussig Cancer Center, Cleveland Clinic, Cleveland, OH 44106, USA; 13 Department of Stem Cell and Immune Regulation, Yokohama City University Graduate School of Medicine, Yokohama 236-0004, Japan; kirino@yokohama-cu.ac.jp; 14 Sorbonne University, 75020 Paris, France; sophie.georgin-lavialle@aphp.fr; 15 Department of Internal Medicine, DMU3ID, Tenon Hospital, 75020 Paris, France; 16 CEREMAIA (French National Reference Center for Autoinflammatory Diseases), 75012 Paris, France; 17 ERN RITA (European Reference Network for Rare Immunodeficiency, Autoinflammatory and Autoimmune Disease Network), 75012 Paris, France; 18 Medical Affairs and Clinical Development, Sobi, Inc., Waltham, MA 02451, USA; sarah.buckley@sobi.com (S.A.B.); raman.garcha@sobi.com (R.G.); bryan.harder@sobi.com (B.G.H.); 19 Department of Internal Medicine, Division of Rheumatology, Mayo Clinic, Rochester, MN 55905, USA; koster.matthew@mayo.edu

**Keywords:** VEXAS, inflammation, pacritinib, clinical trial

## Abstract

VEXAS (Vacuoles, E1 ubiquitin-activating enzyme, X-linked, Autoinflammatory, Somatic) syndrome is a systemic disorder characterized by an overlap of hematologic and inflammatory features. Most patients require chronic use of moderate-to-high doses of glucocorticoids (GCs) to maintain disease control. Data on GC-sparing therapies is limited, and there have been no prospective pharmacotherapeutic trials in VEXAS syndrome published to date. Pacritinib, an oral inhibitor of IRAK1, JAK2, and ACVR1, has emerged as a promising therapeutic option for VEXAS syndrome. The PAXIS trial is the first prospective, randomized pharmacotherapeutic study conducted in this rare and severe disease. Utilizing a novel study design and disease-specific endpoints, the trial will evaluate the efficacy and safety of two dose levels of pacritinib compared with placebo in patients with VEXAS syndrome (NCT06782373, EUCTR: 2024-516347-41-00).

## 1. Introduction

VEXAS (Vacuoles, E1 ubiquitin-activating enzyme, X-linked, Autoinflammatory, Somatic) syndrome is a severe, systemic autoinflammatory disease first described in 2020 [1]. Associated with somatic hematopoietic mutations in the *UBA1* gene on the X chromosome, VEXAS mainly affects older men. Prognosis can be poor, with a reported 5-year survival of 63% [2]. Mortality is driven by disease progression, either due to bone marrow failure or severe inflammation, or complications of immunosuppression, including opportunistic infections [3,4,5,6]. Patients with VEXAS often present with diverse features that overlap with other immunologic and rheumatologic conditions, frequently leading to delayed recognition and diagnosis. International consensus guidelines from the American College of Rheumatology recommend that adult males with high clinical suspicion of VEXAS (unexplained systemic inflammation combined with hematologic abnormalities) receive genetic testing for mutations in the *UBA1* gene [7]. The most affected organs include the skin, lungs, joints, cartilage, and blood vessels [8]. Typical clinical manifestations include cutaneous small vessel vasculitis, auricular and nasal chondritis, arthritis, and pulmonary infiltrates [8]. Patients also frequently experience systemic symptoms such as fever, severe fatigue, night sweats, and unintentional weight loss.

Mutations in the *UBA1* gene, which encodes the E1 ubiquitin-activating enzyme, disrupt the normal ubiquitin–proteasome system of protein degradation, inducing the unfolded protein response (UPR) [1]. UPR activation in myeloid and natural killer cells triggers the induction of gene expression signatures of innate immune signaling, largely mediated by nuclear factor kappa B (NFĸB) transcriptional activation, most likely resulting in the systemic inflammation characteristic of patients with VEXAS [1,9,10,11,12,13,14].

Current treatment options for VEXAS are limited. Glucocorticoids (GCs) are the mainstay of treatment, with most patients requiring long-term use at moderate-to-high daily doses [15]. Allogeneic hematopoietic stem cell transplant (allo-HSCT) may be curative. However, allo-HSCT remains experimental in VEXAS, and only a minority of patients will be transplant-eligible. Thus, most patients will continue to require GC therapy [16].

GC monotherapy, although effective to control VEXAS inflammatory features, is associated with toxicities, including susceptibility to opportunistic infections, adverse metabolic effects (weight gain, glucose intolerance, hyperlipidemia, and hypertension), osteoporosis, neurocognitive effects, and impaired wound healing [17,18,19], leading to increased mortality [20,21]. GC-sparing agents are therefore often used in conjunction with GCs, though none have been studied prospectively in a controlled manner, nor have any been approved for the treatment of VEXAS. Additionally, GCs do not address the bone marrow failure component of VEXAS.

**Figure 1:**
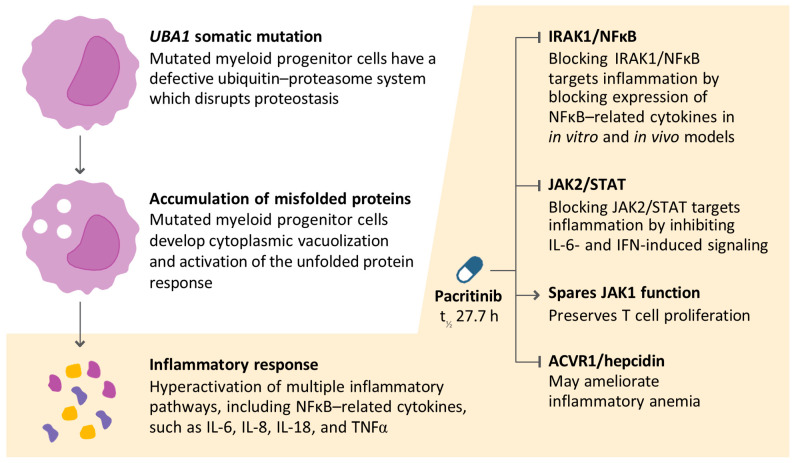
Hypothesized mechanism of action of pacritinib in VEXAS. Pacritinib inhibits three kinases involved in distinct inflammatory signaling cascades induced by somatic hematopoietic mutations in *UBA1* (IRAK1, JAK2, and ACVR1). Pacritinib does not inhibit JAK1 at clinically relevant concentrations. ACVR1, activin A receptor type 1; h, hours; IFN, interferon; IL, interleukin; IRAK1, interleukin 1 receptor-associated kinase 1; JAK, Janus kinase; NFĸB, nuclear factor kappa B; STAT, signal transducer and activator of transcription; t_½_, half-life; TNFα, tumor necrosis factor alpha.

Most GC-sparing strategies in VEXAS have focused on therapies that inhibit individual pro-inflammatory cytokines (e.g., anti-interleukin [IL]-6, anti-IL-1, anti-tumor necrosis factor alpha [TNFα]) or target single inflammatory pathways (e.g., Janus kinase [JAK] inhibitors) [22,23,24]. Despite the important role of NFĸB-induced inflammation in VEXAS, no NFĸB-targeting therapy has been studied in this disease. Pacritinib, an FDA-approved therapy for the treatment of myelofibrosis with severe thrombocytopenia, blocks NFĸB signaling via potent inhibition of IL-1 receptor-associated kinase 1 (IRAK1). In addition, pacritinib blocks JAK2, as well as the hepcidin regulator activin A receptor type 1 (ACVR1) (Figure 1) [25,26]. Pacritinib does not inhibit JAK1 at clinically relevant concentrations, thereby potentially preserving T-cell proliferation and overall hematopoietic cell production compared with dual JAK1/2 inhibitors [27,28]. This selective inhibition profile may be particularly beneficial for patients with VEXAS, in whom maintaining JAK1 activity could support immune function and blood cell recovery.

### 1.1. Rationale and Hypothesis

As a multi-kinase inhibitor that suppresses NFĸB signaling via IRAK1 inhibition, as well as JAK2 and ACVR1, pacritinib has the potential to provide a safe and effective GC-sparing anti-inflammatory option for patients with VEXAS.

### 1.2. Objectives

The primary objective of this study is to evaluate the efficacy of two dose levels of pacritinib compared with placebo during a double-blind treatment period. Secondary objectives, also assessed during a double-blind treatment period, include hematologic improvement, changes in quality of life (QOL), pharmacokinetics (PK), and pharmacodynamics (PD) of each pacritinib dose level compared with placebo. Safety and tolerability will also be assessed to support dose-finding. Exploratory objectives during the double-blind period include changes in disease symptoms, biomarkers, and myeloid mutations in each pacritinib arm compared with placebo.

Additional exploratory objectives assess efficacy and safety during the open-label treatment period, including longer-term outcomes among patients treated with pacritinib throughout the double-blind and open-label periods, as well as outcomes after switching from placebo to pacritinib.

## 2. Materials and Methods

### 2.1. Patient Population

The eligibility criteria for the PAXIS trial are summarized in Table 1. The study will enroll adult patients with inflammatory features due to VEXAS syndrome. A documented pathogenic or likely pathogenic mutation at methionine-41 (M41) or neighboring splice site mutation (c.118-1, c.118-2) in *UBA1* is required. Patients must have current or past inflammatory involvement within 6 months prior to enrollment and be receiving ongoing GC therapy at a dose of 15–45 mg/day (prednisone or prednisolone) for at least 4 consecutive weeks, with stable dosing in the 10 days leading up to enrollment. Patients who previously tapered to 10–14 mg/day following initiation of a non-GC anti-inflammatory therapy may still qualify, provided they had a documented period needing higher doses of GC monotherapy prior to initiation of non-GC anti-inflammatory, the non-GC therapy has been appropriately discontinued (washed out), and the GC dose has been escalated to 15–45 mg/day for symptom control at the time of enrollment. The length of washout varies by agent and was individually established based on either elimination PK (e.g., small molecules) or length of lasting biologic effect after full elimination of the agent (e.g., biologics).

Exclusion criteria include prior allo-HSCT, solid organ transplant, or use of systemic GCs for conditions other than VEXAS syndrome. Patients will be excluded if they have had more than one prior admission to an intensive care unit due to a VEXAS flare within the prior 6 months or if they require ≥9 units of red blood cells (RBC) in the 90 days prior to enrollment. Limiting intensive care unit admissions and high RBC transfusion-dependence would minimize the heterogeneity of disease severity in the study and ensure a more uniform patient population. Patients must not have known concurrent high-risk or very high-risk myelodysplastic syndrome (MDS), MDS requiring antineoplastic treatment or allo-HSCT. Recent malignancy, exposure to hypomethylating agents (HMAs) within 6 months, or receipt of more than 6 cycles of HMAs at any time will also exclude patients from the study.

### 2.2. Study Design

**Figure 2:**
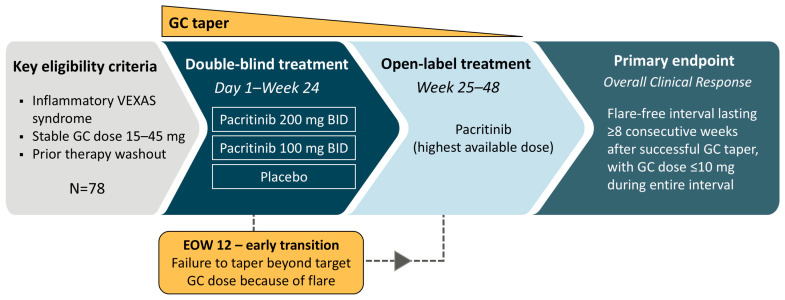
PAXIS trial schema. BID, twice daily; EOW, end of week; GC, glucocorticoid; VEXAS, Vacuoles, E1 ubiquitin-activating enzyme, X-linked, Autoinflammatory, Somatic.

PAXIS (NCT06782373, EUCTR: 2024-516347-41-00) is a randomized, international, multicenter, double-blind, placebo-controlled phase 2 study (Part 1) followed by an open-label treatment period (Part 2) designed to evaluate the efficacy and safety of pacritinib for the prevention of VEXAS flares after GC taper (Figure 2). The study consists of a screening period, a 24-week double-blind treatment period, a 24-week open-label treatment period, and a 30-day post-treatment follow-up period. PAXIS will enroll at 40 sites in 8 countries. This study was approved by the institutional review boards at each institution and conducted in accordance with the principles outlined in the Declaration of Helsinki. All patients will provide written informed consent prior to enrollment in the trial.

There is no standardized or approved treatment for VEXAS syndrome. The design of the PAXIS study is consistent with real-world clinical practice, in which GC taper is routinely used as a method to determine the lowest GC dose at which each patient can remain stable. The safety measures employed in the PAXIS study during the GC taper reflect standard practice, including a slow taper, small taper steps, and weekly check-in calls or study visits prior to each taper step to ensure that taper is medically appropriate. These design features justify the use of both experimental therapy and placebo on the PAXIS trial in conjunction with GC taper.

Approximately 78 patients will be randomized 1:1:1 (26 per arm) to receive pacritinib 200 mg twice daily (BID), pacritinib 100 mg BID plus placebo BID, or placebo BID. Randomization is stratified by prescribed GC dose on the day of randomization (prednisone or prednisolone 15–25 mg vs. >25–45 mg). Patients follow a fixed GC taper while on study, including an 8-week interval at a dose of 10 mg daily, followed by further taper. Patients may receive GC rescue therapy for VEXAS flares.

After 24 weeks of double-blind treatment, all patients will transition to an open-label phase, during which they will receive pacritinib at the highest available dose (i.e., 200 mg BID), unless that dosing arm has been terminated due to futility or safety concerns (as described below).

Patients who experience a disease flare in the first 12 weeks, defined as failure to taper the GC dose to a pre-specified threshold (determined by baseline dose) will be eligible for early transition to open-label treatment at week 12. Once transitioned, patients will receive pacritinib for the remainder of the 48-week study. These patients will be classified as early failures and counted as non-responders in the primary endpoint analysis.

An interim futility and safety analysis will be conducted once 10 patients in each pacritinib arm have completed a minimum of 12 weeks of treatment. Outcomes will be assessed on the two pacritinib arms, and interim results may result in closure of one arm (in which case the study continues with only one pacritinib arm) or both arms (in which case the study is terminated).

### 2.3. Study Endpoints and Outcomes Measures

Efficacy endpoints, including the primary endpoint, are defined according to the response criteria shown in Table 2.

The primary endpoint (assessed during the double-blind period) is overall clinical response (OCR), defined as maintaining a flare-free interval of at least 8 consecutive weeks, while sustaining a GC dose ≤10 mg daily throughout the interval.

The definition of VEXAS flare was established using the Delphi method, based on a systematic review and expert panel consensus, as previously described [29].

Best response during the double-blind period (secondary endpoint) and open-label period (exploratory endpoint) will also be captured and will include more stringent response criteria (including biochemical response requiring normalization of C-reactive protein [CRP]) and less stringent criteria (including partial response and stable disease).

Additional secondary endpoints (assessed during the double-blind period) include number of flare-free days with GC dose <10 mg/day, hematologic improvement in platelets and hemoglobin, change in QOL, and disease symptoms as measured by PROMIS short forms (fatigue, physical function, sleep disturbance), 36-Item Short Form Health Survey (SF-36), and Patient Global Impression of Change (PGIC), as well as PK/PD and safety.

The following exploratory endpoints will be measured during the double-blind portion of the study: QOL and symptom assessments supporting exploratory endpoints include the novel VEXAS-Symptom Assessment Form (VEXAS-SAF) and the EQ-5D-5L. Exploratory measures of disease activity include the novel VEXAS-Disease Activity Index (VEXAS-DAI) [30], as well as the Clinical Global Impression of Change (CGI-C) and Severity (CGI-S). Change in GC toxicity will be measured using the glucocorticoid toxicity index (GTI) [19]. Changes in inflammatory cytokines will be measured, and myeloid mutations, including *UBA1* variant allele frequency (VAF), will be assessed using longitudinal next-generation sequencing (NGS).

The following exploratory endpoints will be measured during the double-blind and open-label portions of the study: Best response, duration of response, hematologic improvement in platelets and hemoglobin, changes in inflammatory cytokines, myeloid mutations, *UBA1* VAF using longitudinal NGS, and changes in GC toxicity using the GTI. These analyses which evaluate the efficacy of pacritinib across the double-blind and open-label treatment periods will be summarized by treatment arm for patients initially randomized to one of the pacritinib treatment arms in the double-blind treatment period.

The following exploratory endpoints will be measured during the open-label period only: Best response, changes in inflammatory cytokines, myeloid mutations, *UBA1* VAF using longitudinal NGS, and changes in GC toxicity using the GTI. These analyses which evaluate the efficacy of pacritinib during the open-label treatment period will be summarized for patients who were initially randomized to placebo in the double-blind treatment period and treated with pacritinib in the open-label treatment period.

### 2.4. Statistical Plan

The planned sample size of 26 patients in each treatment arm would provide >80% power to demonstrate a difference in response rate as defined for the primary endpoint between one of the pacritinib arms and placebo, based on a one-sided Type I error rate of 0.025 for each pacritinib vs. placebo arm pairwise comparison. The overall significance level of one-sided 0.05 is split equally between the two dose comparisons using a Bonferroni correction.

### 2.5. Efficacy Analysis

The analysis will be conducted on the Intention-to-Treat population. The primary efficacy endpoint will be summarized as the percentage of patients who achieve OCR during the double-blind treatment period. Comparisons of OCR rates will be conducted between pacritinib 200 mg BID vs. placebo and pacritinib 100 mg BID vs. placebo, using the stratified Cochran–Mantel–Haenszel test. Stratification will be based on baseline GC dose categories: 15–25 mg vs. >25–45 mg.

All primary and secondary efficacy analyses will be stratified according to the randomization stratification factor (baseline GC dose) to account for its potential influence on outcomes.

### 2.6. Safety Plan and Analysis

The PAXIS trial will be reviewed by an independent data monitoring committee (iDMC) on a regular basis to evaluate the benefit:risk ratio. The iDMC will also perform an interim analysis with futility and safety stopping boundaries for each of the pacritinib arms based on patient data obtained over the first 12 weeks of treatment.

The interim analysis specifies that a pacritinib arm may be terminated for futility if 7 or more of 10 patients meet early failure criteria (failure to taper the GC dose to a pre-specified threshold due to disease flare). A pacritinib arm may be terminated for safety if any of the following criteria are met: 6 or more of 10 patients experience a treatment-related Grade ≥ 3 non-hematological adverse event that is not due to VEXAS flare, laboratory abnormality, or other expected complications; 4 or more of 10 patients experience a treatment-related Grade ≥ 4 infection event; 3 or more of 10 patients experience a treatment-related Grade ≥ 4 bleeding event; 4 or more of 10 patients experience a treatment-related Grade ≥ 3 major adverse cardiovascular event; or 4 or more of 10 patients experience a new thrombotic or thromboembolic event.

Safety results will be summarized in all treated patients.

## 3. Conclusions

The PAXIS trial is the first prospective, randomized study to evaluate a pharmacologic treatment specifically for VEXAS syndrome. It employs a novel study design and disease-specific endpoints. PAXIS may establish pacritinib as an effective treatment for this severe, systemic disease which currently lacks approved therapies. Results from this study will deepen our understanding of VEXAS disease biology, including clarifying the disease’s natural course and the role of NFĸB (and its inhibition) in disease control. Given the heterogeneity of VEXAS syndrome, it is possible that the sample size may be a potential limitation, and a larger sample size would be required to determine whether there are specific patient subsets who are most likely to benefit from pacritinib. There is still limited knowledge of the natural history of the disease, and it is possible for a drug to be more effective than placebo while still not meeting key efficacy endpoints. Ultimately, PAXIS will provide the foundation for future clinical development, refining both clinical endpoints and measurement tools in VEXAS.

## Figures and Tables

**Table 1:** Select eligibility criteria.

Inclusion Criteria	Exclusion Criteria
Documented evidence of a pathogenic mutation (M41 or neighboring splice site [c.118-1, c.118-2]) in *UBA1* Current or documented evidence of past inflammatory involvement within 6 months prior to enrollment Receiving ongoing GC therapy at a stable prednisone or prednisolone dose of 15–45 mg/day * Karnofsky performance status ≥ 50% Absolute neutrophil count ≥ 500/µL Platelet count ≥ 25 × 10^9^/L Peripheral blasts < 5%	Prior allo-HSCT or solid organ transplant (other than corneal) Current use of systemic GCs for conditions other than VEXAS syndrome More than one prior admission to an intensive care unit due to a VEXAS syndrome flare within the prior 6 months Received ≥9 units of red blood cell transfusions in the 90 days prior to enrollment Known concurrent MDS requiring antineoplastic treatment, or allo-HSCT, or known high-risk MDS based on the IPSS-R Malignancy within 1 year prior to enrollment, curatively treated non-melanoma skin cancer, or curatively treated carcinoma in situ. Patients with a pre-malignant hematologic condition (e.g., MGUS, clonal cytopenia of unknown significance) may enroll Exposure to HMA within 6 months prior to enrollment, or exposure to more than 6 cycles of HMAs at any time Exposure to non-GC anti-inflammatory therapies or necessity of hematologic support therapies within protocol defined timeframes prior to enrollment ^#^ Exposure to anti-platelet therapy apart from low-dose aspirin (≤100 mg daily) within 28 days prior to enrollment Grade ≥ 2 bleeding within the prior 3 months, unless precipitated by an inciting event History of clinically significant cardiovascular disease, or clinically significant abnormalities in rhythm or conduction during screening, including: QTc > 480 ms; Grade ≥ 3 cardiac events within the prior 3 months. New thrombosis within the prior 60 days

* Patients who are stable on GC doses of 10–14 mg/day in addition to another non-GC anti-inflammatory therapy screening who have a previously documented VEXAS flare on a GC dose ≥ 10 mg/day may be eligible provided that their GC dose is escalated to 15–45 mg/day after washout. ^#^ Anti-CD20 agents (e.g., rituximab): 180 days; Anti-IL-23 agents (e.g., ustekinumab): 90 days; Anti-TNFα except for etanercept (e.g., infliximab): 60 days; Intravenous anti-IL-6 agents (e.g., tocilizumab): 42 days; Subcutaneous anti-IL-6 agents: 28 days; Anti-IL-17 agents (e.g., secukinumab): 28 days; Anti-integrins (e.g., vedolizumab): 60 days; Intravenous immunoglobulin: 28 days; Danazol, immunomodulatory imide drugs, luspatercept, or thrombopoietin receptor agonists: 28 days; Cytotoxic chemotherapy: 28 days; Etanercept: 21 days; Oral JAK inhibitors: 14 days; Anti-IL-1 agents: Anakinra: 14 days; Canakinumab: 60 days; Any other non-GC anti-inflammatory therapy (e.g., mycophenolate, azathioprine, cyclosporine, sulfasalazine, methotrexate): 14 days. allo-HSCT, allogeneic hematopoietic stem cell transplant; GC, glucocorticoid; HMA, hypomethylating agents; IL, Interleukin; IPSS-R, Revised International Prognostic Scoring System; JAK, Janus kinase; MDS, myelodysplastic syndrome; MGUS, monoclonal gammopathy of unknown significance; VEXAS, Vacuoles, E1 ubiquitin-activating enzyme, X-linked, Autoinflammatory, Somatic.

**Table 2:** Efficacy endpoint thresholds.

	Endpoint Definition	Flare-Free Interval	GC Daily Dose	CRP
**Overall clinical response** (primary endpoint)	Stringent clinical biochemical response	≥8 consecutive weeks	≤5 mg	≤10 mg/L
	Clinical biochemical response	≥8 consecutive weeks	≤10 mg	≤10 mg/L **or** ≥50% reduced from baseline and a value ≤20 mg/L
	Clinical response	≥8 consecutive weeks	≤10 mg	
	Partial clinical response	≥8 consecutive weeks	≥50% reduced from baseline	
	Stable disease	≥8 consecutive weeks	≤baseline	
	Non-response	Not meeting other response criteria		

GC daily dose = glucocorticoid dose required during every day during the flare-free interval. CRP = C-reactive protein level required every day during the flare-free interval.

## Data Availability

The datasets analyzed during the current study are available from the corresponding author on reasonable request. Sobi is committed to responsible and ethical sharing of data on the participant level and summary data for medicines and indications approved by the European Medicines Agency and/or Food and Drug Administration while protecting individual participant integrity and compliance with applicable legislation. Data access will be granted in response to qualified research requests. All requests are evaluated by a cross-functional panel of experts within Sobi and a decision on sharing will be based on the scientific merit and feasibility of the research proposal, maintenance of personal integrity and commitment to publication of the results. To request access to study data, a data sharing request form (available on www.sobi.com) should be sent to medical.info@sobi.com. Further information on Sobi’s data sharing policy and process for requesting access can be found at https://www.sobi.com/en/policies (accessed on 8 February 2026).
